# The link between yeast cell wall porosity and plasma membrane permeability after PEF treatment

**DOI:** 10.1038/s41598-019-51184-y

**Published:** 2019-10-14

**Authors:** Arunas Stirke, Raimonda Celiesiute-Germaniene, Aurelijus Zimkus, Nerija Zurauskiene, Povilas Simonis, Aldas Dervinis, Arunas Ramanavicius, Saulius Balevicius

**Affiliations:** 1 Center for Physical Sciences and Technology, Sauletekio ave. 3, LT-10257 Vilnius, Lithuania; 2 Department of Biochemistry and Biophysics, Life Sciences Center, Sauletekio ave. 7, LT-10257 Vilnius, Lithuania; 3 Department of Physical Chemistry, Faculty of Chemistry and Geosciences, Vilnius University, Naugarduko st. 24, LT-03225 Vilnius, Lithuania

**Keywords:** Biophysics, Biological physics

## Abstract

An investigation of the yeast cell resealing process was performed by studying the absorption of the tetraphenylphosphonium (TPP^+^) ion by the yeast *Saccharomyces cerevisiae*. It was shown that the main barrier for the uptake of such TPP^+^ ions is the cell wall. An increased rate of TPP^+^ absorption after treatment of such cells with a pulsed electric field (PEF) was observed only in intact cells, but not in spheroplasts. The investigation of the uptake of TPP^+^ in PEF treated cells exposed to TPP^+^ for different time intervals also showed the dependence of the absorption rate on the PEF strength. The modelling of the TPP^+^ uptake recovery has also shown that the characteristic decay time of the non-equilibrium (PEF induced) pores was approximately a few tens of seconds and this did not depend on the PEF strength. A further investigation of such cell membrane recovery process using a florescent SYTOX Green nucleic acid stain dye also showed that such membrane resealing takes place over a time that is like that occurring in the cell wall. It was thus concluded that the similar characteristic lifetimes of the non-equilibrium pores in the cell wall and membrane after exposure  to  PEF indicate a strong coupling between these parts of the cell.

## Introduction

The molecules, which enter cells such as yeast or bacteria, must pass through the cell wall, a periplasmic space and then obtain access to the transporters located in the plasma membrane. The porosity of the yeast cell wall is highly regulated and is dependent on the cell cycle. This is important for water retention in the periplasmic space, where some important proteins, such invertase^1^, alpha galactosidase^2^ and acid trehalase^3^ are located. The cell wall is considered to be a sieve-like structure and does not present an absolute obstacle for solutes since it is being highly charged negatively by the phosphate and carboxyl groups in the cell wall with mananns playing the role of an ion exchanger^4^. Hence, the cell wall acts as the first selective and primary barrier for nutrients, biomolecules and ions^5^. It isn’t clear, but there is some evidence that molecules such as bleomycin and tetraphenylphosphonium may also be suspended by this biosorption process^6–9^. There are many ways to regulate (mainly to increase) the rate at which these various molecules can pass the cell wall barrier. This can be done by using chemical treatment with organic solvents and detergents^10^ or by mechanical shearing or treatment with a pulsed electric field (PEF), thus generating pores^11^.

The other very important barrier for molecules diffusion is the plasma membrane. Besides simple diffusion, when gasses and small lipophilic molecules are being transported, yeast cells have many instruments (transporters and channels) to preserve cell homeostasis^12^. This is the reason why the lipid bilayer is always confronted with hyper osmotic pressure at the interior of the cell due to the impermeable intracellular compounds. This osmotic pressure is the main mechanical force that stretches the plasma membrane and this can be at times the reason for cell lysis^13^. It is known that there exists a relationship between the turgor pressure and cell wall pathway integrity^14^. Moreover, there is evidence that the plasma membrane fluidity changes the mechanical properties of the cell wall due to the fact that some of the cell wall proteins are linked to the membrane via the GPI (glycosylphosphatidylinositol) anchor^15^. However, the actual molecular mechanism of such a connection is not clear.

Our previous work demonstrated that the permeability to the lipophilic ion tetraphenylphosphonium (TPP^+^) across the yeast cell membrane and cell wall may be reversibly changed by their exposure to PEF of microsecond duration^8^. Moreover, the accumulation of these TPP^+^ ions by the yeast is not affected by an electric field when they reach a steady-state after 90–120 min, while exposure to a high (≈100 kV/cm) nanosecond duration PEF speeds this process up to 65 times^16^. As far as we know, the influence of post-pulse resealing on the elevated TPP^+^ absorption rate has not been investigated. Moreover, as was noted, the duration and character of the transient pore decay is essential for understanding the mechanism of PEF induced yeast cell permeabilization^17^.

In this paper, we investigated yeast cell electrical permeabilization by applying a PEF regime, which does not affect the viability of the cells, i.e. most of the cells can recover to their initial (non-permeabilized) state. These investigations were performed by applying two different methods. One method was based on the study of the TPP^+^ ion absorption kinetics using a potentiometric ion selective electrode, which provides data about the yeast cell wall permeabilization. Another method, based on fluorescent dye measurements, showed the permeabilized state of the cell membrane. These experimental results were analyzed by applying the mathematical model, which took into consideration the decay of the post-pulse transient pores. Experiments and modeling have shown a strong link between the yeast cell membrane and the wall resealing process after PEF action.

## Materials and Methods

### Yeast strain and cultivation

*Saccharomyces cerevisiae* SEY6210 (*MATα, leu2-3, leu2-112, ura3-52, his3-* Δ*2*00*, trp1-*Δ*9*0*1, lys2-8*0*1, suc2-*Δ*9, GAL*) yeast cells were grown to their early exponential growth phase in a complete medium (YPD) containing 1% (w/v) yeast extract, 2% (w/v) peptone and 2% (w/v) glucose (Merck, Darmstadt, Germany). The yeast cells were then re-suspended in 1 ml of a cold (4 °C) electroporation buffer (EPB) containing 1 M sorbitol and a 20 mM Tris-HCl buffer, pH 7.4 (Applichem, Darmstadt, Germany) while keeping the final concentration of the yeasts at the (4–6) × 10^9^ colony forming unit (CFU)/mL. Cell density was determined by measuring the optical density (OD) using an optical absorption spectrophotometer (Halo RB-10, Dynamica Scientific, UK) operating at a wavelength of 600 nm.

### Spheroplasts preparation

After the modification cells were pelleted, washed twice and suspended in a lyticase specific buffer, additional control samples of non-modified cells were prepared in parallel. The lyticase buffer containing 1.2 M of sorbitol, 0.5 mM of MgCl_2_ and 35 mM of K_2_HPO_4_ (pH 6.0) was adjusted for this solution according to our previous report^18^. The final optical density of the diluted suspension was 1.5 OD (600 nm). A further 50 U/ml of lyticase were added to each cell sample and then incubated at 30 °C for 2 hours. To prove that the lyticase enzyme was active during the experiment, its optical density was measured at 600 nm before and after the incubation cycles of the sample suspensions. After such digestion, both cell samples were inspected with a bright field optical microscope. For spheroplasts preparation, the yeast cells (≈1 · 10^7^ cells/ml) were suspended and washed with sterile water. After that, the cells were harvested by centrifugation and resuspended in a SCEM buffer (1 M sorbitol, 0.1 M sodium citrate (pH = 5.6), 10 mM EDTA and 30 mM 2-mercaptoethanol (Merck KGaA, Germany)). Then 30 U/mL of lyticase (Sigma-Aldrich, USA) was added for digestion by the cell walls. After a 2-hour incubation at 30 °C with occasional inversions, the cells were then collected, washed and re-suspended using a STC buffer (1 M sorbitol, 10 mM Tris-HCl (pH = 7.5), 10 mM CaCl_2_ (Merck KGaA, Germany)) by centrifugation at 200·g 5 min.

### Pulsed electric field treatment technique

A programmable electroporator for the generation of high-voltage square-wave electric pulses (developed at the Center for Physical Sciences and Technology, Vilnius, Lithuania) was used for the PEF experiments. This electroporator has a variable energy storage capacitor, thus accumulation of high energy is avoided. It generates a single or a sequence of single electrical pulses with widths from 3 μs to 10 ms and voltages up to 3.5 kV. It has an LCD screen for the monitoring of the input parameters and an additional LCD screen for the display of the pulse waveform (the current passing through the cuvette with the yeast sample) during the experiment. When experiments are performed with a 2 mm gap electroporation cuvette (VWR International, Taiwan) connected to the device, the electric field strength across the cuvette can reach up to about 16 kV/cm.

### Cell wall permeability measurements

For the investigation of the permeability of the yeast cell wall, the concentration changes of TPP^+^ ions as a function of time was measured. A custom-build mini-potentiostat with a special high sensitivity electronic circuit, protected against static electrical interference, was used for the measurements^19^. The electrode with the selective membrane was fabricated according to the protocol found in the literature^20^. During all measurements of the changes of the lipophilic tetraphenylphosphonium (TPP^+^) ion concentrations, the TPP^+^ chloride (Acros Organics, Belgium) was used. For all the measurements, the intact yeast cell and spheroplasts suspension concentrations were the 4–6 × 10^9^ colony forming unit (CFU)/mL. For the evaluation of the changes of the TPP^+^ concentration in both yeast suspensions, 200 μL of the yeast suspension was added to 2 mL of a 1 μM concentration TPP^+^ solution and the potentiometric changes were recorded. The concentration of TPP^+^ ions was calculated from the calibration curve^19^.

To evaluate the role of the cell wall and cell membrane in the TPP^+^ absorption process, square waveform electrical pulses of 150 μs duration and amplitudes of electric field strength (*E*) (up to 4.2 kV/cm) were applied to the intact yeast cells and spheroplasts.

To investigate the resealing of the yeast cell walls after such PEF treatment, the TPP^+^ was added to the electroporation cuvette with the yeast suspension. The initial TPP^+^ concentration (*N*_*is*_) of 1 μM was added to the cuvette at different time intervals (Δ*t* = 5, 10, 20, 30, 40, 50, 60, 80, 120 and 180 seconds) after the PEF treatment (*E* = 5.85, 4.38 and 2.93 kV/cm, *t*_p_ = 150 μs). It was then incubated for *t* = 3 min. at 20 °C in each case and centrifuged in order to get cell free supernatant. These experiments were repeated three times and presented as means values. The quantity of TPP^+^ (*N*) absorbed by the yeast cells (*N* = *N*_*is*_ − [TPP^+^]_supernatant_) was expressed as the accumulation ratio *N/N*_*m*_, where *N*_*m*_ is the maximal concentration of TPP^+^ that can be accumulated in yeast^8^.

### Investigation of the resealing of the cell membrane

The resealing of the cell membrane after treatment with PEF (square waveform electric pulses with amplitude of 5.85 kV/cm and duration of 150 μs) was evaluated using fluorescent dye^21^ (by loading the cell with Fluorescent SYTOX Green nucleic acid stain (Thermofisher Scientific, USA)). During the investigation, the yeast cell suspension was kept on ice. The fluorescent dye was added to the suspension of up to a 125 nM final concentration at different time intervals in the range from 10 s to 600 s after exposure to the PEF. After incubation for one minute, the specimen was placed into the fluorescence measurements cuvette (light path of 1 cm) of spectrometer LS50B (Perkin Elmer, USA) and exposed to 505 ± 5 nm wavelength light. The cell membrane permeability was evaluated as the fluorescence spectrum intensity at a peak wavelength equal to 529 nm.

## Results

### TPP^+^ absorption by yeast cells

**Figure 1:**
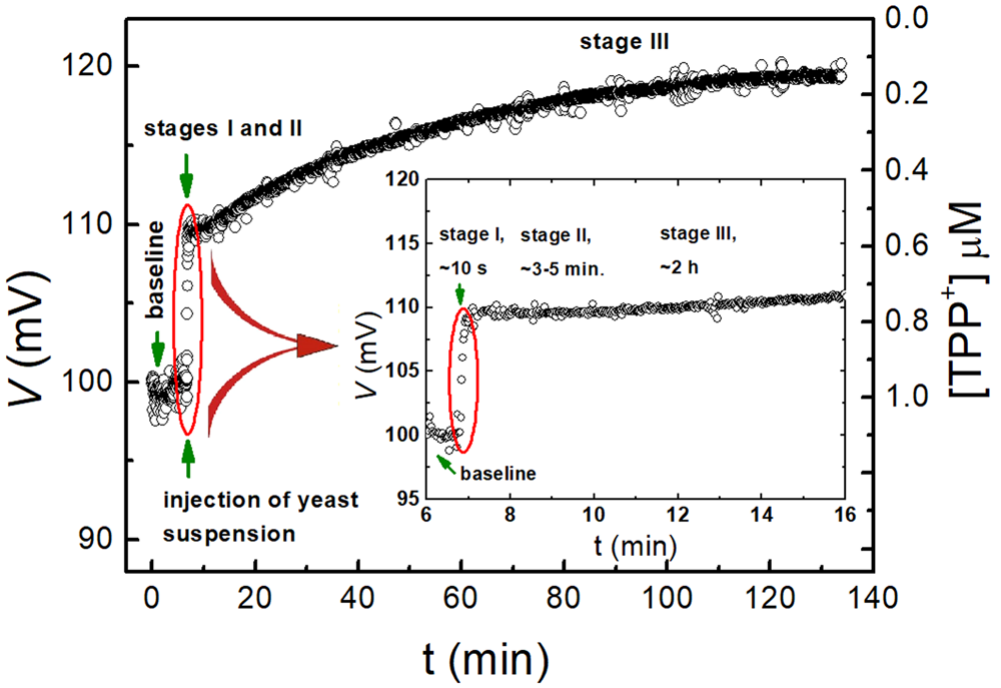
The response signal from the TPP^+^ selective electrode *vs*. time dependence in the yeast suspension. The stages of the TPP^+^ ions absorption kinetics are outlined in the inset.

The ability of the yeast cells to accumulate the TPP^+^ ions was investigated by measuring the kinetics of the TPP^+^ absorption by the cells not treated with the electric field. For this purpose, 200 μL of the yeast suspension was injected into a 2 mL solution having different concentrations of TPP^+^ ions. The typical signal of the response from the TPP^+^ selective electrode *vs*. time dependence reflecting the amount of absorbed TPP^+^ ions is shown in Fig. 1. As it can be seen, the TPP^+^ ions uptake kinetics consisted of three stages. These were (i) stage I, lasting ~10 s. This was the fast stage, which appeared immediately after the injection of the yeast cells into the TPP^+^ solution; (ii) stage II, or the delay stage, which lasted approximately 3–5 minutes after the fast stage, when the concentration of the TPP^+^ changes became no more than 10% (see inset in Fig. 1) and (iii) stage III, when the slow TPP^+^ absorption took place and saturation was reached after ~2 hours.

### PEF influence on TPP^+^ absorption

**Figure 2:**
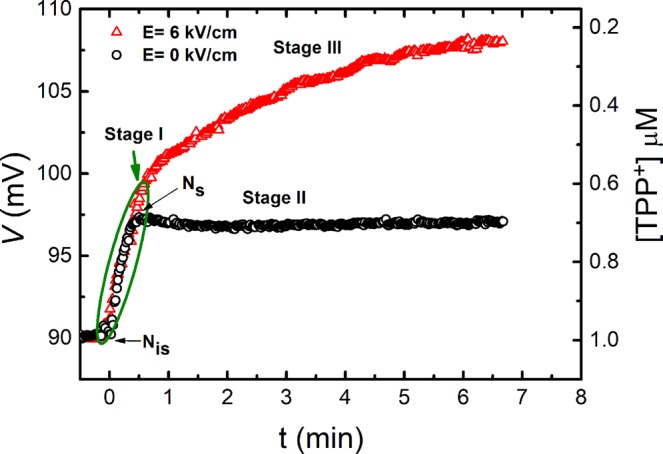
The response of the signal the from TPP^+^ selective electrode *vs*. time dependence in the yeast suspension, when E = 0 kV/cm (circles), and 6 kV/cm (triangles).

The influence of the PEF on the kinetics of the TPP^+^ uptake is shown in Fig. 2. The circular symbols (black) in Fig. 2 correspond to the TPP^+^ absorption by the yeast cells, which were not affected by the PEF and all show the three TPP^+^ uptake stages as was described in previous section. The triangular symbols (red) show the kinetics of the TPP^+^ absorption by the yeast after exposure to PEF of 6 kV/cm strength and 150 μs duration. As can be seen in the figure, stage II, i.e. the delay of the TPP^+^ uptake is absent and the fast TPP^+^ concentration decrease (response to the voltage increase corresponding to stage I), is changed to a slower one (stage III). In both cases, (the cells affected and not affected by the PEF), the fast stage was of the same rate, but of a slightly different amplitude. It was thus obvious that the PEF significantly changes the TPP^+^ absorption rate, i.e. it eliminates the delay stage.

### TPP^+^ absorption by spheroplasts

**Figure 3:**
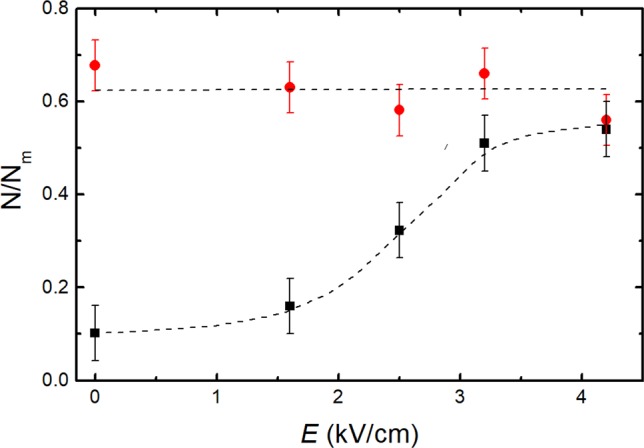
A comparison of the relative change of the TPP^+^ concentration (*N*) normalized to the maximal value (*N*_m_) accumulated by intact yeast cells (square dots) and wall-free spheroplasts (circular dots) 3 min. after PEF treatment (150 μs pulse duration, up to 4.2 kV/cm electric field strength).

For a comparison of the PEF action on intact yeast cells containing wall and wall-free spheroplasts, the TPP^+^ absorption measurements were performed at different electric field strengths. Figure 3 presents the ratio *N*/*N*_m_ dependence on *E*, where *N* is the TPP^+^ amount accumulated by yeast cells after a 3 min. exposure to 1 µM of TPP^+^.

As it can be seen from Fig. 3, the influx of TPP^+^ in the case of the intact yeast increased with an increase of the PEF strength. However, there were no changes of the TPP^+^ influx in the case of wall-free spheroplasts. It is thus evident that PEF can affect the TPP^+^ influx only in the yeast cells having walls and that the cell walls are mainly responsible for the changes of the TPP^+^ uptake rates of the yeast cells.

### The recovery of cell membrane and wall barrier function after PEF

Our previous investigations demonstrated that high amplitude PEF is able to increase the rate of TPP^+^ ions uptake by several times and that saturation is reached in relatively short period of time. As it was found that PEF amplitude not exceeding 6 kV/cm with pulse duration of 150 μs does not affect yeast cells viability^8^, thus these PEF parameters were applied in this study.

### Cell membrane resealing after PEF

**Figure 4:**
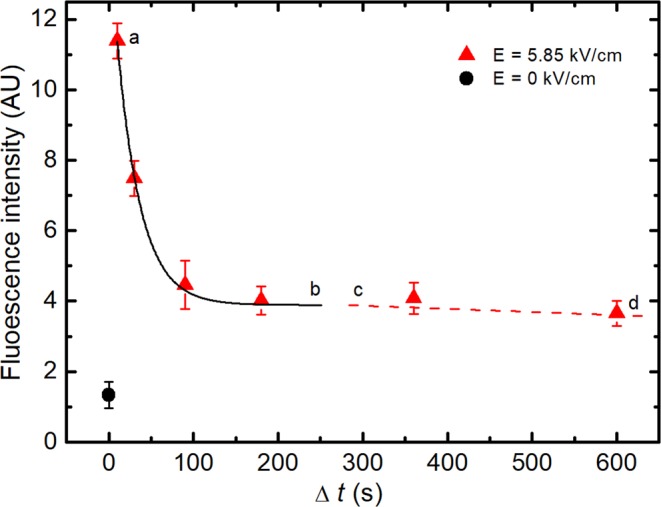
Fluorescence spectrum peak (at 529 nm) intensity *vs*. time (Δ*t*) of the dye added to the cells after PEF treatment (triangles). The black circle shows the intensity of the fluorescence in the case of the control (untreated) cells. The solid curve (**a**,**b**) obtained using Eq. (1), adj. R^2^ is 0.99394. The dashed line (**c**,**d**) represents the extended membrane resealing processes.

To study the permeability of the cell membrane after treatment with PEF, the fluorescent dye SYTOX Green was used. The fluorescent dye was added to the cell suspension with different time delays (Δ*t* = 10, 30, 90, 180, 360 and 600 seconds) after treating the cells with PEF (150 μs pulse duration 5.85 kV/cm electric field strength). Figure 4 shows the intensity of the fluorescent spectrum peak value (529 nm) *vs*. time (Δ*t*) of the fluorescent dye added to the cells after PEF.

The experimental data presented in Fig. 4 show that the fluorescence intensity drops with the increase of time after cell treatment by PEF down to the level of the residual intensity (*I*_r_). The total decay time showing cell membrane relaxation to their initial state after PEF action is approximately 100 seconds and the fluorescence intensity decay can be well fitted by the exponential expression:

1 $$I={I}_{0}\cdot \exp (-\,\frac{\Delta t}{{\tau }_{l}})+{I}_{r}$$

here *I*_0_ is the fluorescence intensity at Δ*t* = 0 and *τ*_*l*_ is the characteristic decay time of the lipidic pores. This model describes that part of the resealing process, where the change of fluorescence intensity is fast (see Fig. 4 solid curve a,b). The fitting results show that permeability of a significant part of the cell membrane recovers to its initial state in a characteristic time *τ*_*l*_ ≈ 20 s. The difference between fluorescence intensity of untreated cells (Fig. 4, black circle) and the residual intensity (Fig. 4, dashed line c,d) might be related to the extended membrane resealing processes due to the experimental setup where the cells were kept on ice^22^.

### Cell wall recovery after PEF

**Figure 5:**
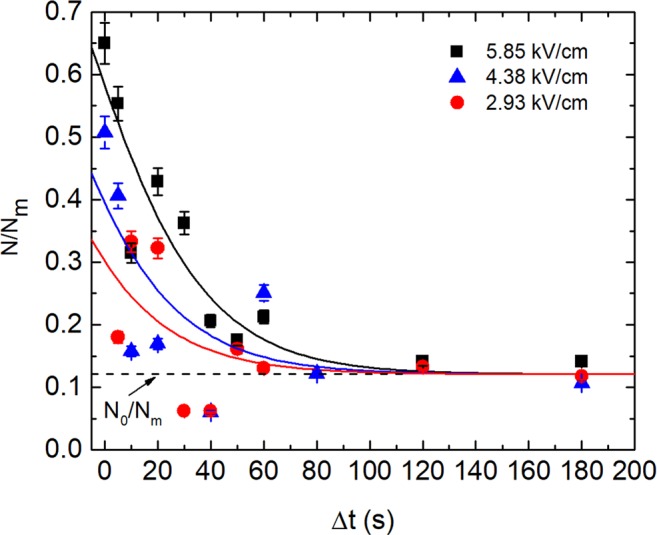
The dependence of TPP^+^ absorption by yeast cells over time after PEF treatment. The y axis represents the relative TPP^+^ amount accumulated in the yeast cells; the x axis represents the TPP^+^ injection time after PEF treatment. The dashed line represents the full recovery of the TPP^+^ absorption level by the yeast cells (PEF = 0) after 3 minutes of incubation. The solid curves were obtained by using Eq. (10), adj. R^2^ values are 0.854 (rectangles), 0.582 (triangles) and 0.341 (circles).

The recovery of the barrier function of the cells walls was investigated by analyzing the TPP^+^ absorption process of the yeast cells after PEF action with pulse amplitudes of three different electric field strengths. The results of this investigation are presented in Fig. 5.

Here *N*/*N*_m_ is the ratio between the TPP^+^ amount (*N*) accumulated inside the yeast cells after 3 min. exposure in a TPP^+^ solution and the maximal amount (*N*_m_) of TPP^+^ which can be accumulated in the tested volume of yeast (without PEF action). The dashed line (*N*_0_/*N*_m_) represents the full recovery of the TPP^+^ absorption process which corresponds to the relative amount of TPP^+^ (*N*_0_/*N*_m_ ≈ 0.12) absorbed during 3 min. of incubation. With the rise of the electric field strength from 2.93 kV/cm to 5.85 kV/cm, the relative TPP^+^ amount absorbed by cells drops to the full recovery level in approximately 90–110 seconds. The solid curves in Fig. 5 represent fitting results using model described in the next Section. It has to be noted that the characteristic time evaluated from modelling results was obtained similar for all three electric field strengths.

### The modelling of cell wall recovery process after PEF

The experimental results presented in Fig. 5 show that the recovery of the yeast cell wall after PEF action takes place in a relatively short time when compared to the duration of the whole 1.5 h-long TPP^+^ ions absorption process (without PEF) until the steady state is reached (see Fig. 1). This means that the post-pulse transient pores in cell wall responsible for the increase of TPP^+^ absorption rate exist only for a few minutes after the electric pulse action. At high electric field strength (more than 6 kV/cm), the TPP^+^ ions uptake by the yeast significantly increases and saturation is reached in a relatively short time^8^. In such case, the TPP^+^ ions absorption process was described while ignoring the cell wall recovery after the PEF action. Thus for the description of TPP^+^ absorption kinetics, the following pseudo-second order differential equation was used^8^:

2 $$\frac{dN(E,t)}{dt}={k}_{a}(E,t)\cdot ({N}_{m}-N)({N}_{s}-N);$$

here *N*(t) is the time dependent concentration of the TPP^+^ ions in the supernatant, *N*_m_ is maximal change of the TPP^+^ concentration during the slow period of TPP^+^ accumulation by the yeast and *N*_s_ is the concentration of TPP^+^ ions at the beginning of stage II (corresponding to TPP^+^ concentration at the time instant when the absorption process starts (see Fig. 2)), *k*_α_ is absorption rate coefficient. It was assumed, that *k*_α_ depends on the electric field strength as follows^8^:

3 $${k}_{\alpha }={k}_{\alpha 0}+b{({E}_{p}-{E}_{th.p})}^{2}{f}^{2}{[1-\exp (\frac{{t}_{p}}{{t}_{ch}})]}^{2};$$

here *b* is the empirical constant, which shows the effectiveness of the electric field on permeabilization, *f* is the cell shape factor (1.5 for spheres), *E*_p_ is the amplitude of electric field strength, *E*_th.p_ is the threshold electric field strength at which the TPP^+^ absorption rate begins to be dependent on the electric field, *t*_p_ is the pulse duration and *t*_ch_ is the characteristic time required to charge the membrane as a capacitor.

However, at lower amplitudes of electric field strength (less than 6 kV/cm in our case), the decay of the post-pulse transient pores in the cell walls can significantly change the kinetics of the TPP^+^ ions adsorption by the yeast cells. For this reason, the coefficient *k*_a_ in Eq. (2) was modified by considering that it was not only a function of the pulsed electric field strength and duration, but also depended on the resealing of the transient pores after PEF action.

In order to obtain a mathematical expression of coefficient’s *k*_a_ dependence on time, we made the following assumptions: (a) that the PEF permeabilization which facilitated the TPP^+^ ions diffusion is proportional to the number of pores in the cell wall; (b) that the generation of the additional post-pulse non-equilibrium pores is the result of PEF action and that the number of these pores depends on the PEF energy (amplitude and duration of the square waveform pulses); (c) that the characteristic lifetime of the post-pulse transient non-equilibrium pores and the naturally existing equilibrium pores is different. Based on these assumptions the following expression of *k*_a_ was suggested:

4 $${k}_{a}(E,t)={k}_{a0}+C\cdot \Delta {M}_{p}(E,t);$$

here *k*_a0_ is the absorption coefficient at *E* = 0 kV/cm, Δ*M*_p_(*E*, *t*) = *M*_p_ − *M*_p,eq_ is the difference between the whole amount of post-pulse transient non-equilibrium pores (*M*_p_) and the amount of naturally existing equilibrium pores (*M*_p,eq_), and *C* is a constant. The dependence of Δ*M*_p_ on time can be obtained from the following first order differential equation:

5 $$\frac{d(\Delta {M}_{p})}{dt}=-\,k\cdot (\Delta {M}_{p});$$

here *k* = 1/*τ*_d_ (*τ*_d_ is the characteristic lifetime of the PEF induced pore). The solution of the Eq. (5) is the following:

6 $$\Delta {M}_{p}(t)=\Delta {M}_{p0}\cdot {e}^{-\frac{t}{{\tau }_{d}}}\,;$$

here Δ*M*_p0_ is the initial amount of post-pulse transient non-equilibrium pores generated after the PEF action. Taking into account Eqs (3, 4), one can express Δ*M*_p0_ as a function of electric field strength:

7 $$\Delta {M}_{p0}=\frac{b}{C}{({E}_{p}-{E}_{th.p})}^{2}{f}^{2}\,{[1-\exp (-\frac{{t}_{p}}{{t}_{ch}})]}^{2}.$$

The solution of Eq. (2) using Eqs (4) and (6) is the following:

8 $$N(t)=\frac{{N}_{m}(1-S)}{1-R\cdot S};$$

9 $${\rm{here}}\,R=\frac{{N}_{m}}{{N}_{s}}\,{\rm{and}}\,S={e}^{({N}_{m}-{N}_{s})[{k}_{a0}\cdot t+C\cdot \Delta {M}_{p0}\cdot {\tau }_{d}(1-{e}^{-\frac{t}{{\tau }_{d}}})]}.$$

We considered the TPP^+^ ions absorption delay at the time intervals Δ*t* after the PEF action (Fig. 5). This could be described by Eq. (8) using modified expression (9). It has to be noted that Eqs (8 and 9) represent the solution of Eq. (2) in which the time *t* starts from the moment when TPP^+^ was added to the cuvette with the yeast suspension. In order to investigate the absorption delay, an experiment was performed by adding TPP^+^ with a Δ*t* delay after PEF action. Therefore, Δ*M*_p0_ in Eq. (9) had to be replaced by Δ*M*_p0_ exp(−Δt/τ_d_), because the concentration of the non-equilibrium pores had exponentially decreased during the time Δ*t*. In such case, the *S* in Eq. (9) is modified as follows:

10 $${S}_{\Delta t}={e}^{({N}_{m}-{N}_{s})[{k}_{a0}\cdot t+C\cdot \Delta {M}_{p0}\cdot {\tau }_{d}({e}^{-\frac{\Delta t}{{\tau }_{d}}})(1-{e}^{-\frac{t}{{\tau }_{d}}})]}.$$

The solid curves in Fig. 5 show the fitting of the experimental data using modified Eq. (8), when *S*Δ_*t*_ instead of *S* (see Eq. (9)) was used:

11 $$\frac{N(t,\Delta t)}{{N}_{m}}=\frac{1-{S}_{\Delta t}}{1-R\cdot {S}_{\Delta t}}.$$

During fitting the incubation time *t*, after which the absorption of the TPP^+^ was measured, was kept constant *t* = 3 min. The level *N*_*0*_*/N*_*m*_ marked by the dashed line in Fig. 5 corresponds to the full recovery of the cell wall, where *N*_*0*_ is the amount of the TPP^+^ absorbed by untreated yeast cells:

12 $$\frac{{N}_{0}}{{N}_{m}}=\frac{1-{S}_{0}}{1-R\cdot {S}_{0}};$$

here *S*_0_ is:

13 $${S}_{0}={e}^{({N}_{m}-{N}_{s})[{k}_{a0}\cdot t]}.$$

Thus, the *N/N*_*m*_ − *N*_*0*_ /*N*_*m*_ in Fig. 5 shows the difference between the TPP^+^ amount absorbed by the PEF affected cells (*N*) at Δ*t* and the amount of TPP^+^ absorbed by the untreated yeast cells (*N*_*0*_) relative to maximal amount *N*_*m*_. The data when Δ*t* = 0 in Fig. 5 represent the *N/N*_*m*_ ratio immediately after the PEF action. One can see that at Δ*t = 0* for *E* = 5.85 kV/cm, the ratio *N/N*_*m*_ ≈ 0.65, which is in good agreement with the results presented in^8^, where the absorption coefficient’s *k*_a_ dependence on *E* was expressed as Eq. (3) without considering its dependence on delay time Δ*t*. The fitting results using this model (solid curves in Fig. 5) show that the characteristic time (τ_d_) of the wall recovery almost does not depend on the PEF electric field strength. Due to scatter of experimental data the fitting procedure resulted in *τ*_*d*_ values in the range of (21–27) s, thus the solid curves in Fig. 5 were shown for average *τ*_*d*_ = 24 s.

## Discussion

Tetraphenylphosphonium (TPP^+^), a lipophilic cation is widely used as a probe for various cells as well as an artificial membrane potential measurements^20,23–26^. The ability of the aromatic groups to delocalize and shield the electric charge and to increase the lipid solubility facilitates their translocation across the hydrophobic barrier^27,28^. The accumulation of TPP^+^ ions in yeast cells strongly depends on various factors, such as the yeast strain, the properties of the cell wall, the yeast growth phase, the growing conditions and mutations determining the structure of the cell wall and others^25,29–31^. Unlike structurally similar compound dibenzyldimethylammonium, TPP^+^ is not transported via the thiamine transport system, nor via another inducible translocation mechanism. As described in the literature, the uptake of TPP^+^ ions into the yeast cells has distinct stages. Two groups of authors, Eraso *et al*. and Slayman *et al*. observed the biphasic uptake of TPP^+^ when the yeast was collected in the logarithmic phase of growth^25,32^. In both cases, the exponential and quasi-linear stages were outlined. Ketteter *et al*. experimented with artificial lipid bilayer membranes, which showed a three-stage uptake: (1) adsorption to the membrane-solution interface; (2) passage of the ion to the opposite interface; and (3) desorption into the aqueous solution. The rate-determining step is the jump from the adsorption site into the aqueous phase^24^. For lipophilic molecules such as TPP^+^, it usually takes from 0.5 up to 2 or more hours to reach the steady state^25,32^. In our experiments, three stages of TPP^+^ uptake was observed (Fig. 1). Stage I was due to the injection of yeast suspension into the sample; stage II, when the TPP^+^ concentration changes were delayed for several minutes. As far as we know, these were absent in the works of other authors due to the different experimental setups. The last stage, i.e. stage III, consisting of slow TPP^+^ absorption and a plateau, is similar to biphasic TPP^+^ absorption, which is outlined in the works of Eraso *et al*. and Slayman *et al*.^25,32^.

Stage I in our experiments could be explained by TPP^+^ ions adsorption on the surfaces of negatively charged yeast cells^4^ and the interaction between it and the positive TPP^+^ ions (i.e. sorbate). A similar behavior of the sorbate was observed during the investigation of the yeast cells, when the fast, reversible and metabolically-independent adsorption step was attributed to the cell wall surface complexation with metal cation uptake^33^. The stage II, i.e. delay stage, could be explained by the additional time needed for the TPP^+^ ions to reach the surface of the plasma membrane through the cell wall and periplasmic space. The slow stage reflects the process of TPP^+^ absorption deeper inside the yeast cell and through the membrane. It should be noted that due to the large TPP^+^ concentration at the surface of the cell, the expulsion of TPP^+^ ions from the cell to the solution has to be negligible. Thus, the yeast cells accumulate TPP^+^ ions mainly during the last slow stage. As is known, the main ion transport systems into the yeast cell include ion pumps, several transporters, a potassium channel and a non-selective cation current. The presence of a complex structure cell wall adds more complexity for ion transport. Thus, the TPP^+^ absorption experiments were performed in cell wall-free spheroplasts, which were obtained by degrading the wall with the enzyme lyticase. Such spheroplasts provide an easy means to manipulate the experimental system for studies of whole genome transferring^34^, cell fusion^35^ or toxin transport^36^. In our experiments, the system of spheroplasts served for the clarification of the role of the cell wall in TPP^+^ absorption. As seen from Fig. 3 (circular dots), the PEF strength had no effect on TPP^+^ absorption for spheroplasts, which was almost the same at all applied electric field strengths including *E* = 0. This result showed that the main barrier for TPP^+^ ion absorption is the cell wall, not the membrane.

**Scheme 1:**
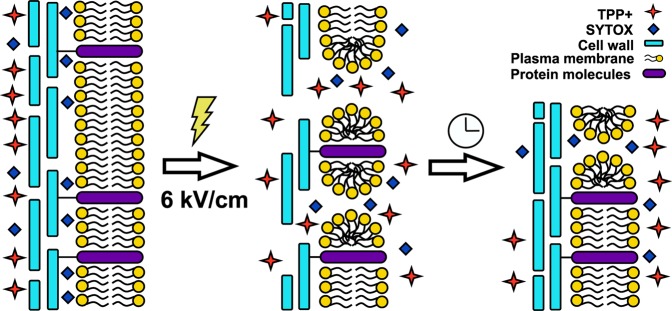
Schematic overview of the cell wall and the resealing of the cell membrane after the PEF treatment.

The experimental results presented in Figs 4 and 5 demonstrated that both the membrane and the wall relaxation process after PEF lasted for approximately 100 s. Moreover, the characteristic time of these relaxations evaluated by means of mathematical simulations was also of the same order. Good fitting was also obtained at all the different electric field strengths, which had been applied, of the same characteristic lifetime of the post-pulse transient pores in the cell walls. This was *τ*_d_ = 24 s, which was close enough to the characteristic lifetime of lipidic pores, which was *τ*_*l*_ ≈ 20 s (see Fig. 4). However, as is known from molecular dynamics related with thermal processes, the post-pulse lipidic pore lifetime is in the range from 100 ns^17^ to 3·ms^37,38^, which is drastically shorter than the characteristic relaxation time observed in our experiments. Moreover, experimental investigation showed that this relaxation time could range from milliseconds^39^ to several hours if the cells are cooled down after electroporation by putting them on ice^22^. It is evident, that the lifetimes of the lipid pores may vary along a broad range and depend on the experimental setup or the object^40^. In our case, however, this cannot be attributed to the pores, which disappear via spontaneous thermal fluctuations. Yeast treatment with PEF simultaneously induces permeabilization of the whole cell barrier, i.e. both the cell wall and the membrane. The recovery of these parts to a non-permeabilized state takes a similar time (Scheme 1).

The strong connection between the resealing of the yeast cell membrane and the wall recovery allows one to assume that this recovery process is “mechanical” in nature, which could be related to changes of osmotic pressure during electroporation and the processes taking place after the PEF. The verification of this hypothesis could be an object of future investigations.

In conclusion, our investigations confirmed that the rate of absorption of the TPP^+^ ions into the yeast cells is stimulated by the action of the pulsed electric field (PEF). Moreover, as the main barrier to the influx of TPP^+^ ions is the cell wall and the PEF stimulates the generation of transient (non-equilibrium) pores in this wall, this is responsible for the increase of the TPP^+^ ions absorption rate. It was demonstrated that the treatment of the yeast by the PEF simultaneously induces permeabilization of both parts of this cell barrier, i.e. the cell wall and the membrane. The characteristic time of recovery of these parts to a non-permeabilized state takes several tens of seconds, what demonstrates a strong coupling between these processes.
